# Crystal structure and Hirshfeld surface analysis of lapachol acetate 80 years after its first synthesis

**DOI:** 10.1107/S2056989019011393

**Published:** 2019-08-19

**Authors:** Miguel A. Martínez-Cabrera, Mario A. Macías, Francisco Ferreira, Enrique Pandolfi, Javier Barúa, Leopoldo Suescun

**Affiliations:** a Universidad Nacional de Asunción, Facultad de Ciencias Exactas y Naturales, Departamento de Biología, Área Química Orgánica de los Productos Naturales-LAREV, San Lorenzo Campus-UNA, Paraguay; b Department of Chemistry, Universidad de los Andes, Cra 1 N° 18A-12, 111711, Bogotá, Colombia; c Universidad Nacional de Asunción, Facultad de Ciencias Exactas y Naturales, Laboratorio de Análisis Instrumental, Departamento de Química, San Lorenzo Campus-UNA, Paraguay; d Laboratorio de Síntesis Orgánica, DQO, Facultad de Química, Universidad de la República, Montevideo 11800, Uruguay; e Universidad Nacional de Asunción, Facultad de Ciencias Químicas, San Lorenzo Campus-UNA, Paraguay; f Cryssmat-Lab/DETEMA, Facultad de Química, Universidad de la República, Av. Gral. Flores 2124, Montevideo 11800, Uruguay

**Keywords:** crystal structure, naphtho­quinone, lapachol acetate, synthesis, Hirshfeld analysis

## Abstract

Lapachol acetate [systematic name: 3-(3-methyl­but-2-en­yl)-1,4-dioxonaph­thalen-2-yl acetate], C_17_H_16_O_4_, was prepared using a modified high-yield procedure and its crystal structure is reported for the first time 80 years after its first synthesis. The full spectroscopic characterization of the mol­ecule is reported. The mol­ecular conformation shows little difference with other lapachol derivatives and lapachol itself. The packing is directed by inter­molecular π–π and C—H⋯O inter­actions, as described by Hirshfeld surface analysis. The former inter­actions make the largest contributions to the total packing energy in a ratio of 2:1 with respect to the latter.

## Abstract 2

A modified synthesis procedure allowed lapachol acetate (acetic acid 3-(3-methyl-but-2-en­yl)-1,4-dioxo-1,4-di­hydro- naphthalen-2-yl ester) to be obtained in high yield and its crystal structure is reported for the first time 80 years after its first synthesis. The lapachol acetate mol­ecular conformation is very similar to that of reported lapachol mol­ecules and other derivatives. The monoclinic *P*2_1_/*n* crystal structure packs through weak inter­molecular π–π and C—H⋯O inter­actions as described by Hirshfeld surface analysis.

## Chemical context   

Naphto­quinones are natural products characterized by a naphthalene ring system exhibiting a *para*-quinone motif in positions 1,4. They are natural pigments and normally substituted by hydroxyl or methyl groups or present as glycosides (Bruneton, 2001 ▸). Among the natural products, they possess remarkable biological activity such as anti­bacterial, anti­fungal, anti­parasitic, anti­viral and anti­cancer (Babula *et al.*, 2007 ▸; da Silva & Ferreira, 2016 ▸; Miranda *et al.*, 2019 ▸; Araújo *et al.*, 2019 ▸; Barbosa Coitinho *et al.*, 2019 ▸; Strauch *et al.*, 2019 ▸). Lapachol (2-hy­droxy-3-(3-methyl-but-2-en­yl)[1,4]naphtho­quinone), isolated from *Handroanthus Heptaphyllus* (Vell.) Mattos, a native species from Paraguay, was studied as a hemisynthetic precursor of lapachol acetate {[3-(3-methyl-but-2-en­yl)-1,4-dioxonaphthalen-2-yl]acetate} for the first time by Cooke *et al.* (1939 ▸). Jacobsen & Torsell (1973 ▸) prepared lapachol acetate using 2-acet­oxy-1,4-naphtho­quinone as a precursor with 79% yield. We developed an optimization of the first synthesis of lapachol acetate developed by Cooke *et al.* (1939 ▸), introducing several modifications with the purpose of standardizing it and increasing the yield to 97.5%. Details of the synthesis and the spectroscopic characterization are included in the supporting information. Noting that the crystal structure of lapachol acetate had not been reported, we also undertook the crystallization and structure determination.

## Structural commentary   

**Figure 1:**
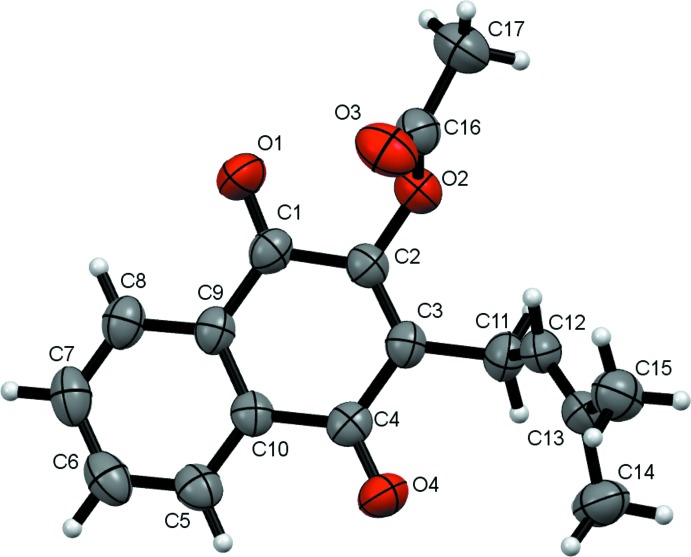
*ORTEP* view of a lapachol acetate mol­ecule with the labelling scheme, and displacement ellipsoids drawn at the 50% probability level. One of the two positions of the disordered C17 methyl group has been omitted for clarity.

**Figure 2:**
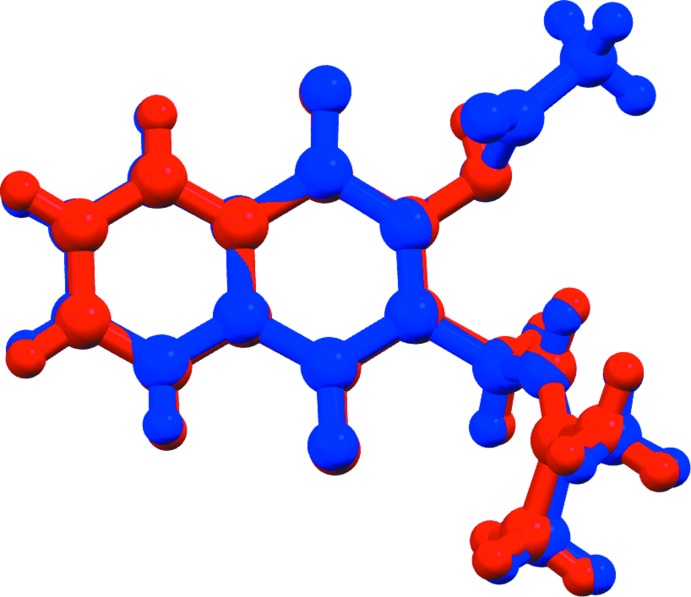
Overlay of a lapachol acetate (blue) and a lapachol (red) mol­ecule (LAPA II mol­ecule as described by Larsen *et al.*, 1992 ▸). Only the common C/O atoms were fitted.

The lapachol acetate mol­ecule (Fig. 1 ▸) is the ester of lapachol at the alcohol moiety (O2 in Larsen *et al.*, 1992 ▸). The mol­ecule is composed of three planar groups, the naptho­quinone nucleus comprising atoms C1 to C11, O1, O2 and O4, and two smaller butenyl and acetate residues at the sides. The butenyl and acetate mean planes are inclined to the naphthoquinone mean plane by 65.80 (10) and 78.52 (11)°, respectively. The lapachol acetate mol­ecule shows typical bond distances and angles, and overlaps very closely with the common part of the lapachol mol­ecule in the structure LAPA II reported by Larsen *et al.* (1992 ▸) (Fig. 2 ▸), with an average deviation of C/O atomic positions of 0.158 Å and a maximum deviation of 0.309 Å for atom O4. This is rather unexpected, since the butenyl moiety shows rotational flexibility around the C3—C11 and C11—C12 bonds. However, in both reported lapachol polymorphs (Larsen *et al.*, 1992 ▸) and two derivatives (Eyong *et al.*, 2015 ▸; da Silva *et al.*, 2012 ▸) reported in the CSD (Groom *et al.*, 2016 ▸) with the same rotational freedom, the dihedral angle between the planar C=C(CH_3_)_2_ group and the naphtho­quinone nucleus is close to 70°, as observed in lapachol acetate.
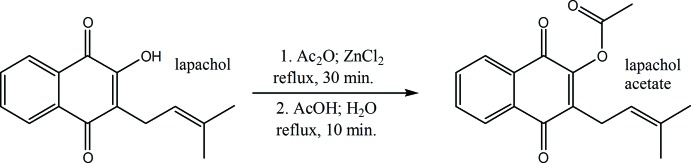


## Supra­molecular features   

**Figure 3:**
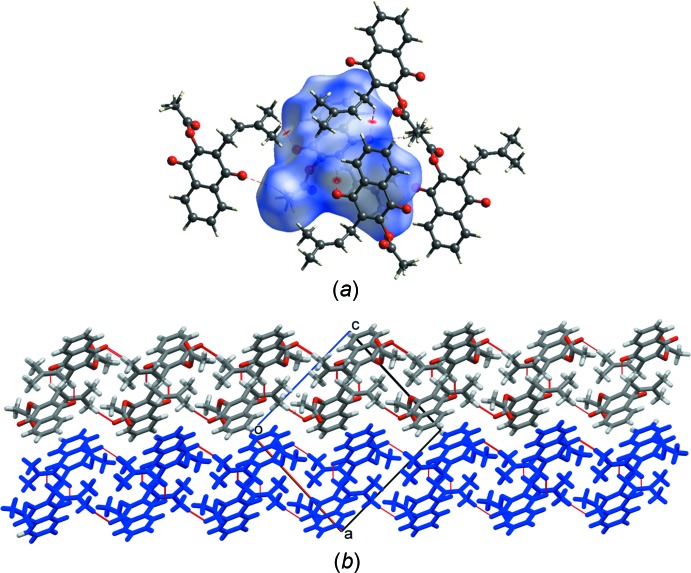
(*a*) View of the Hirshfeld surface for lapachol acetate mapped over *d*_norm_ showing the C—H⋯O hydrogen-bond inter­actions. (*b*) The mol­ecular structure of lapachol acetate showing the formation of stacked (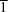
01) sheets.

Crystals of lapachol acetate are held together by weak dipolar and dispersion forces because there is no strong H-donor residue in the mol­ecule. Mol­ecules connect with other units through weak C(*sp^3^*)—H⋯O=C hydrogen bonds H11*B*⋯O4^i^ and H15*A*⋯O1^ii^ (Table 1 ▸, Fig. S4*a* in the supporting information) defining double sheets of mol­ecules parallel to (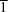
01). The butenyl residue of a screw-rotation-related mol­ecule adds an inter­molecular π–π inter­action with the naphto­quinone residue to the sheets with atoms C12^iii^ and C13^iii^ located at 3.243 and 3.544 Å, respectively, from the naphto­quinone plane (Fig. S4*b*). Finally, the double sheets stack along the [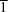
01] direction where naphtho­quinone nuclei of inversion-related mol­ecules display π–π inter­actions. Ring 1 (C1–C4/C10/C9; centroid *Cg*1) of the mol­ecule is close to ring 2 (C5—C10; centroid *Cg*2), the *Cg*2⋯*Cg*1^iv^ distance being 3.8532 (12) Å); *Cg*2⋯*Cg*2^iv^ is the shortest distance [3.8035 (13) Å], with an average perpendicular distance between naphto­quinone planes of 3.3787 (9) Å, as shown in Fig. S4*c* [symmetry codes: (i) −*x* + 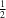, *y* − 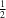, −*z* + 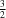; (ii) *x* + 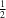, −*y* + 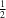, *z* + 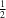 *;* (iii) 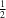 − *x*, 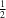 + *y*, 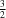 − *z*; (iv) 1 − *x*, 1 − *y*, 1 − *z*). Considering the Hirshfeld (HF) surface (Turner *et al.*, 2017 ▸) mapped over *d*_norm_ (analysis of the contact distances *d*_i_ and *d*_e_ from the HF surface to the nearest atom inside and outside, respectively), these inter­actions in one mol­ecule are shown in Fig. 3 ▸ *a* and the sheets of mol­ecules defined by them in Fig. 3 ▸ *b*. The 2D fingerprints of lapachol acetate (shown in Fig. S5 of the supporting information) show no particular features other than the aforementioned H⋯O/O⋯H contacts, which comprise 28.2% of the total HF surface, revealing their importance in the formation of the crystal.

**Figure 4:**
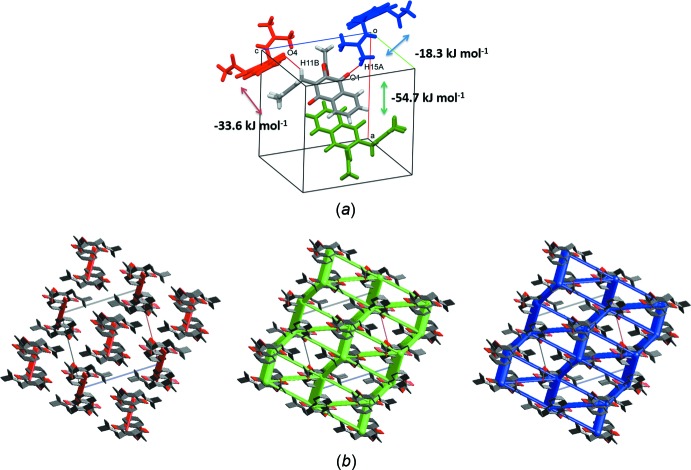
(*a*) Mol­ecular close contacts and (*b*) energy-framework diagrams for electrostatic (red) and dispersion (green) contributions to the total inter­action energy (blue) in lapachol acetate crystals.

In order to describe these inter­actions in a whole-of-mol­ecule approach, accurate model energies of the inter­actions between mol­ecules of lapachol acetate in the crystal were analysed. The inter­actions were calculated using the B3LYP/6–31 G(d,p) energy model implemented in *CrystalExplorer* (Turner *et al.*, 2017 ▸), which uses quantum mechanical charge distributions for unperturbed mol­ecules (Mackenzie *et al.*, 2017 ▸). In the calculations, the total energy is modelled as the sum of the electrostatic (*E*_ele_), polarization (*E*_pol_), dispersion (*E*_dis_) and exchange-repulsion (*E*_rep_) terms (Mackenzie *et al.*, 2017 ▸). The strongest pairwise inter­action with a total energy of −54.7 kJ mol^−1^ corresponds to the inter­action between neighbouring aromatic systems, while the mol­ecules connected through a combination of π–π inter­actions between the butenyl and naphto­quinone residues and C11—H11*B*⋯O4^i^ hydrogen bonds have a total energy of −33.6 kJ mol^−1^. The weakest inter­action, C15–H15*A*⋯O1^ii^, shows a total energy of −18.3 kJ mol^−1^ (Fig. 4 ▸ *a*). The energy framework diagrams for lapachol acetate (Fig. 4 ▸ *b*) show that electrostatic forces act to keep pairs of inversion-related chains of mol­ecules joined while dispersion forces act in three dimensions to build the crystal structure. The total energy diagram shows a high resemblance to the dispersion framework, showing that these forces are the most important in the crystal. The inter­action energies for selected mol­ecular pairs in the first coordination sphere around the asymmetric unit are summarized in Table S1 and Fig. S6 of the supporting information.

## Database survey   

Lapachol and its derivatives are rather scarce in the Cambridge Crystal Structure Database (Version 5.40, update 2 of May 2019; Groom *et al.*, 2016 ▸) with only 31 entries matching the basic C—O framework of lapachol. Two lapachol polymorphs LAPA I and LAPA II (Larsen *et al.*, 1992 ▸) have been reported at 105 K, as mentioned above. Two additional lapachol derivatives obtained by replacing one H atom have been reported during the current decade: 4-(3-hy­droxy-1,4-dioxo-1,4-di­hydro­naphthalen-2-yl)-2-methyl­but-2-enal (Eyong *et al.*, 2015 ▸) is an aldehyde of lapachol at C15 and 3-(3-methyl­but-2-en-1-yl)-1,4-dioxo-1,4-di­hydro­naphthalen-2-yl 4-methyl­benzene­sulfonate (Silva *et al.*, 2012 ▸) is a sulfonate at O2. Lapachol acetate is the third derivative of this kind reported. Some lapachol derivatives where cyclization of the 3-methy-2-butenyl moiety or coordination with metals (as lapacholate) have additionally been reported, but are not related to lapachol acetate.

## Synthesis and crystallization   

Lapachol was obtained from an extract of *Handroanthus Heptaphyllus* (Vell.) Mattos, the pink trumpet tree (or lapacho negro) as described in the supporting information. 201 mg (0.823 mmol) of lapachol were dissolved in 5 ml of dry acetic anhydride and a catalytic amount of dry zinc chloride (ZnCl_2_) was added. The suspension was refluxed for 30 min under an inert atmosphere (N_2_). The solution was allowed to cool and 5 ml of glacial acetic acid and later 50 ml of distilled water were added. The final mixture was refluxed for 10 min and allowed to precipitate overnight. The crude solid was filtered, dried and purified by column chromatography (hexa­ne: AcOEt, 9: 1 *v*/*v*) to obtain pure lapachol acetate as yellow crystals (see the detailed description of the obtention of lapachol and the synthesis of lapachol acetate in the supporting information and the detailed spectroscopic study in Figs. S1–S3). Adequate crystals for diffraction were obtained by dissolving a few mg of the solid in a minimum amount of AcOEt in a rubber-stop vial with a syringe needle through the center to promote slow evaporation of the solvent at room temperature.

## Refinement   

Crystal data, data collection and structure refinement details are summarized in Table 2 ▸. H atoms were placed in calculated positions (C—H = 0.93–0.97 Å) and included as riding contributions, with isotropic displacement parameters set at 1.2–1.5 times the *U*_eq_ value of the parent atom. The C17 methyl group shows rotational disorder that was modelled with two positions that were refined with a fixed C—H bond distance but with rotational freedom (AFIX 147) converging at occupancies of 0.79 (3) and 0.21 (3).

## Supplementary Material

Crystal structure: contains datablock(s) I. DOI: 10.1107/S2056989019011393/ex2023sup1.cif

Structure factors: contains datablock(s) I. DOI: 10.1107/S2056989019011393/ex2023Isup2.hkl

Details of synthesis, spectroscopic characterization and intermolecular interactions of lapachol acetate. DOI: 10.1107/S2056989019011393/ex2023sup4.pdf

Supporting information file. DOI: 10.1107/S2056989019011393/ex2023Isup3.cml

CCDC reference: 1947085

Additional supporting information:  crystallographic information; 3D view; checkCIF report

## Figures and Tables

**Table 1:** Hydrogen-bond geometry (Å, °)

*D*—H⋯*A*	*D*—H	H⋯*A*	*D*⋯*A*	*D*—H⋯*A*
C11—H11*B*⋯O4^i^	0.97	2.55	3.274 (3)	131
C15—H15*A*⋯O1^ii^	0.96	2.59	3.485 (3)	156

Symmetry codes: (i) 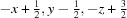; (ii) 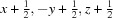.

**Table 2:** Experimental details

Crystal data	
Chemical formula	C_17_H_16_O_4_
*M*_r_	284.30
Crystal system, space group	Monoclinic, *P*2_1_/*n*
Temperature (K)	296
*a*, *b*, *c* (Å)	12.0914 (8), 9.4741 (6), 12.7761 (9)
β (°)	92.943 (4)
*V* (Å^3^)	1461.64 (17)
*Z*	4
Radiation type	Cu *K*α
μ (mm^−1^)	0.75
Crystal size (mm)	0.26 × 0.22 × 0.18

Data collection	
Diffractometer	Bruker D8 Venture/Photon 100 CMOS
Absorption correction	Multi-scan (*SADABS*; Krause *et al.*, 2015 ▸)
*T*_min_, *T*_max_	0.654, 0.754
No. of measured, independent and observed [*I* > 2σ(*I*)] reflections	7496, 2996, 1972
*R*_int_	0.038
(sin θ/λ)_max_ (Å^−1^)	0.637

Refinement	
*R*[*F* ^2^ > 2σ(*F* ^2^)], *wR*(*F* ^2^), *S*	0.048, 0.136, 1.03
No. of reflections	2996
No. of parameters	193
H-atom treatment	H-atom parameters constrained
Δρ_max_, Δρ_min_ (e Å^−3^)	0.17, −0.16

Computer programs: *APEX2* and *SAINT* (Bruker, 2014 ▸), *SHELXT* (Sheldrick, 2015*a* ▸), *SHELXL2018* (Sheldrick, 2015*b* ▸), *Mercury* (Macrae *et al.*, 2008 ▸) and *publCIF* (Westrip, 2010 ▸).

## supplementary crystallographic information

### Crystal data

**Table:**

C_17_H_16_O_4_	*D*_x_ = 1.292 Mg m^−^^3^
*M**_r_* = 284.30	Melting point: 352(1) K
Monoclinic, *P*2_1_/*n*	Cu *K*α radiation, λ = 1.54178 Å
*a* = 12.0914 (8) Å	Cell parameters from 7958 reflections
*b* = 9.4741 (6) Å	θ = 4.9–75.7°
*c* = 12.7761 (9) Å	µ = 0.75 mm^−^^1^
β = 92.943 (4)°	*T* = 296 K
*V* = 1461.64 (17) Å^3^	Block, yellow
*Z* = 4	0.26 × 0.22 × 0.18 mm
*F*(000) = 600	

### Data collection

**Table:**

Bruker D8 Venture/Photon 100 CMOS diffractometer	2996 independent reflections
Radiation source: Cu Incoatec microsource	1972 reflections with *I* > 2σ(*I*)
Detector resolution: 10.4167 pixels mm^-1^	*R*_int_ = 0.038
\j and ω scans	θ_max_ = 79.2°, θ_min_ = 4.9°
Absorption correction: multi-scan (SADABS; Krause *et al.*, 2015)	*h* = −13→15
*T*_min_ = 0.654, *T*_max_ = 0.754	*k* = −11→9
7496 measured reflections	*l* = −16→15

### Refinement

**Table:**

Refinement on *F*^2^	Primary atom site location: dual
Least-squares matrix: full	Secondary atom site location: difference Fourier map
*R*[*F*^2^ > 2σ(*F*^2^)] = 0.048	Hydrogen site location: inferred from neighbouring sites
*wR*(*F*^2^) = 0.136	H-atom parameters constrained
*S* = 1.03	*w* = 1/[σ^2^(*F*_o_^2^) + (0.053*P*)^2^ + 0.3463*P*] where *P* = (*F*_o_^2^ + 2*F*_c_^2^)/3
2996 reflections	(Δ/σ)_max_ < 0.001
193 parameters	Δρ_max_ = 0.17 e Å^−^^3^
0 restraints	Δρ_min_ = −0.16 e Å^−^^3^

### Special details

**Table:**

Geometry. All esds (except the esd in the dihedral angle between two l.s. planes) are estimated using the full covariance matrix. The cell esds are taken into account individually in the estimation of esds in distances, angles and torsion angles; correlations between esds in cell parameters are only used when they are defined by crystal symmetry. An approximate (isotropic) treatment of cell esds is used for estimating esds involving l.s. planes.

### Fractional atomic coordinates and isotropic or equivalent isotropic displacement parameters (Å^2^)

**Table:**

	*x*	*y*	*z*	*U*_iso_*/*U*_eq_	Occ. (<1)
O1	0.22182 (14)	0.34297 (18)	0.34233 (12)	0.0686 (5)	
C1	0.25877 (15)	0.3726 (2)	0.42998 (15)	0.0468 (5)	
O2	0.17186 (11)	0.16221 (16)	0.49500 (11)	0.0540 (4)	
C2	0.24117 (15)	0.2761 (2)	0.51890 (15)	0.0441 (5)	
O3	0.30987 (14)	0.05735 (18)	0.41410 (15)	0.0756 (5)	
C3	0.28099 (15)	0.2973 (2)	0.61720 (15)	0.0443 (5)	
O4	0.39859 (12)	0.43627 (17)	0.72673 (11)	0.0586 (4)	
C4	0.35332 (15)	0.4218 (2)	0.64003 (14)	0.0440 (5)	
C5	0.42753 (17)	0.6488 (2)	0.57604 (17)	0.0529 (5)	
H5	0.460252	0.663841	0.642578	0.063*	
C6	0.43917 (18)	0.7485 (3)	0.49821 (19)	0.0615 (6)	
H6	0.479948	0.830043	0.512619	0.074*	
C7	0.39054 (19)	0.7275 (3)	0.39920 (19)	0.0612 (6)	
H7	0.397581	0.795506	0.347504	0.073*	
C8	0.33163 (17)	0.6057 (3)	0.37709 (17)	0.0547 (5)	
H8	0.299507	0.591249	0.310242	0.066*	
C9	0.32006 (15)	0.5041 (2)	0.45465 (15)	0.0444 (5)	
C10	0.36737 (15)	0.5268 (2)	0.55524 (15)	0.0426 (4)	
C11	0.26046 (18)	0.1969 (2)	0.70541 (16)	0.0533 (5)	
H11A	0.260239	0.249275	0.770631	0.064*	
H11B	0.188017	0.154157	0.693426	0.064*	
C12	0.34630 (17)	0.0828 (2)	0.71541 (16)	0.0505 (5)	
H12	0.350981	0.023269	0.657923	0.061*	
C13	0.41622 (17)	0.0566 (2)	0.79619 (16)	0.0510 (5)	
C14	0.4219 (2)	0.1431 (3)	0.89505 (18)	0.0719 (7)	
H14A	0.412225	0.082669	0.954178	0.108*	
H14B	0.364369	0.213071	0.891358	0.108*	
H14C	0.492685	0.188880	0.902682	0.108*	
C15	0.4952 (2)	−0.0652 (3)	0.7946 (2)	0.0684 (7)	
H15A	0.569682	−0.031304	0.805937	0.103*	
H15B	0.487694	−0.111606	0.727755	0.103*	
H15C	0.478890	−0.130827	0.848916	0.103*	
C16	0.2148 (2)	0.0591 (2)	0.43364 (17)	0.0557 (5)	
C17	0.1276 (2)	−0.0443 (3)	0.3994 (2)	0.0785 (8)	0.79 (3)
H17A	0.072901	0.001798	0.354279	0.118*	0.79 (3)
H17B	0.093164	−0.081079	0.459726	0.118*	0.79 (3)
H17C	0.160520	−0.120203	0.362194	0.118*	0.79 (3)
C17'	0.1276 (2)	−0.0443 (3)	0.3994 (2)	0.0785 (8)	0.21 (3)
H17D	0.058095	−0.016088	0.425921	0.118*	0.21 (3)
H17E	0.147328	−0.136138	0.426025	0.118*	0.21 (3)
H17F	0.121161	−0.047259	0.324252	0.118*	0.21 (3)

### Atomic displacement parameters (Å^2^)

**Table:**

	*U*^11^	*U*^22^	*U*^33^	*U*^12^	*U*^13^	*U*^23^
O1	0.0903 (12)	0.0652 (12)	0.0484 (8)	−0.0047 (9)	−0.0162 (8)	0.0054 (8)
C1	0.0471 (10)	0.0480 (12)	0.0449 (11)	0.0085 (9)	−0.0009 (8)	0.0029 (9)
O2	0.0536 (8)	0.0488 (9)	0.0596 (8)	−0.0054 (7)	0.0025 (7)	−0.0014 (7)
C2	0.0430 (9)	0.0407 (11)	0.0486 (10)	0.0036 (9)	0.0025 (8)	0.0002 (9)
O3	0.0664 (10)	0.0607 (12)	0.1004 (13)	0.0026 (9)	0.0100 (9)	−0.0191 (10)
C3	0.0449 (10)	0.0430 (12)	0.0456 (10)	0.0058 (9)	0.0060 (8)	0.0037 (9)
O4	0.0653 (9)	0.0646 (11)	0.0452 (8)	0.0030 (8)	−0.0055 (7)	−0.0027 (7)
C4	0.0431 (10)	0.0448 (12)	0.0443 (10)	0.0105 (9)	0.0051 (8)	−0.0028 (9)
C5	0.0557 (12)	0.0456 (13)	0.0581 (12)	0.0034 (10)	0.0095 (10)	−0.0063 (11)
C6	0.0621 (13)	0.0424 (14)	0.0813 (16)	−0.0016 (11)	0.0171 (12)	−0.0029 (12)
C7	0.0673 (14)	0.0464 (14)	0.0715 (15)	0.0066 (11)	0.0198 (12)	0.0138 (12)
C8	0.0571 (12)	0.0534 (14)	0.0539 (12)	0.0109 (11)	0.0061 (9)	0.0098 (11)
C9	0.0457 (10)	0.0428 (12)	0.0452 (10)	0.0088 (9)	0.0065 (8)	0.0049 (9)
C10	0.0431 (9)	0.0373 (11)	0.0479 (10)	0.0086 (8)	0.0078 (8)	−0.0009 (9)
C11	0.0566 (12)	0.0559 (14)	0.0479 (11)	−0.0022 (10)	0.0067 (9)	0.0092 (10)
C12	0.0625 (12)	0.0431 (13)	0.0458 (10)	−0.0042 (10)	0.0010 (9)	0.0028 (9)
C13	0.0543 (11)	0.0490 (13)	0.0494 (11)	−0.0102 (10)	−0.0005 (9)	0.0040 (10)
C14	0.0739 (15)	0.086 (2)	0.0545 (13)	−0.0051 (14)	−0.0078 (11)	−0.0076 (13)
C15	0.0697 (14)	0.0583 (16)	0.0756 (15)	0.0019 (13)	−0.0109 (12)	0.0043 (13)
C16	0.0658 (13)	0.0453 (13)	0.0556 (12)	−0.0005 (11)	−0.0019 (10)	0.0025 (10)
C17	0.0874 (18)	0.0623 (18)	0.0851 (18)	−0.0195 (14)	−0.0028 (14)	−0.0071 (15)
C17'	0.0874 (18)	0.0623 (18)	0.0851 (18)	−0.0195 (14)	−0.0028 (14)	−0.0071 (15)

### Geometric parameters (Å, º)

**Table:**

O1—C1	1.217 (2)	C11—C12	1.500 (3)
C1—C9	1.476 (3)	C11—H11A	0.9700
C1—C2	1.482 (3)	C11—H11B	0.9700
O2—C16	1.371 (3)	C12—C13	1.324 (3)
O2—C2	1.391 (2)	C12—H12	0.9300
C2—C3	1.337 (3)	C13—C15	1.499 (3)
O3—C16	1.189 (3)	C13—C14	1.504 (3)
C3—C4	1.489 (3)	C14—H14A	0.9600
C3—C11	1.505 (3)	C14—H14B	0.9600
O4—C4	1.218 (2)	C14—H14C	0.9600
C4—C10	1.487 (3)	C15—H15A	0.9600
C5—C6	1.384 (3)	C15—H15B	0.9600
C5—C10	1.384 (3)	C15—H15C	0.9600
C5—H5	0.9300	C16—C17'	1.488 (3)
C6—C7	1.382 (3)	C16—C17	1.488 (3)
C6—H6	0.9300	C17—H17A	0.9600
C7—C8	1.377 (3)	C17—H17B	0.9600
C7—H7	0.9300	C17—H17C	0.9600
C8—C9	1.394 (3)	C17'—H17D	0.9600
C8—H8	0.9300	C17'—H17E	0.9600
C9—C10	1.396 (3)	C17'—H17F	0.9600
			
O1—C1—C9	123.21 (19)	H11A—C11—H11B	107.9
O1—C1—C2	120.2 (2)	C13—C12—C11	127.8 (2)
C9—C1—C2	116.57 (16)	C13—C12—H12	116.1
C16—O2—C2	115.91 (16)	C11—C12—H12	116.1
C3—C2—O2	120.42 (18)	C12—C13—C15	121.0 (2)
C3—C2—C1	124.68 (19)	C12—C13—C14	123.5 (2)
O2—C2—C1	114.76 (16)	C15—C13—C14	115.45 (19)
C2—C3—C4	118.84 (18)	C13—C14—H14A	109.5
C2—C3—C11	122.93 (19)	C13—C14—H14B	109.5
C4—C3—C11	118.16 (17)	H14A—C14—H14B	109.5
O4—C4—C10	121.63 (19)	C13—C14—H14C	109.5
O4—C4—C3	119.97 (19)	H14A—C14—H14C	109.5
C10—C4—C3	118.40 (16)	H14B—C14—H14C	109.5
C6—C5—C10	120.2 (2)	C13—C15—H15A	109.5
C6—C5—H5	119.9	C13—C15—H15B	109.5
C10—C5—H5	119.9	H15A—C15—H15B	109.5
C7—C6—C5	120.4 (2)	C13—C15—H15C	109.5
C7—C6—H6	119.8	H15A—C15—H15C	109.5
C5—C6—H6	119.8	H15B—C15—H15C	109.5
C8—C7—C6	120.0 (2)	O3—C16—O2	121.9 (2)
C8—C7—H7	120.0	O3—C16—C17'	127.4 (2)
C6—C7—H7	120.0	O2—C16—C17'	110.7 (2)
C7—C8—C9	120.2 (2)	O3—C16—C17	127.4 (2)
C7—C8—H8	119.9	O2—C16—C17	110.7 (2)
C9—C8—H8	119.9	C16—C17—H17A	109.5
C8—C9—C10	119.8 (2)	C16—C17—H17B	109.5
C8—C9—C1	119.93 (18)	H17A—C17—H17B	109.5
C10—C9—C1	120.30 (18)	C16—C17—H17C	109.5
C5—C10—C9	119.48 (19)	H17A—C17—H17C	109.5
C5—C10—C4	119.83 (18)	H17B—C17—H17C	109.5
C9—C10—C4	120.69 (19)	C16—C17'—H17D	109.5
C12—C11—C3	112.29 (17)	C16—C17'—H17E	109.5
C12—C11—H11A	109.1	H17D—C17'—H17E	109.5
C3—C11—H11A	109.1	C16—C17'—H17F	109.5
C12—C11—H11B	109.1	H17D—C17'—H17F	109.5
C3—C11—H11B	109.1	H17E—C17'—H17F	109.5
			
C16—O2—C2—C3	−112.3 (2)	O1—C1—C9—C10	−175.23 (19)
C16—O2—C2—C1	71.8 (2)	C2—C1—C9—C10	6.1 (3)
O1—C1—C2—C3	178.0 (2)	C6—C5—C10—C9	1.0 (3)
C9—C1—C2—C3	−3.2 (3)	C6—C5—C10—C4	−178.70 (18)
O1—C1—C2—O2	−6.2 (3)	C8—C9—C10—C5	−1.5 (3)
C9—C1—C2—O2	172.54 (16)	C1—C9—C10—C5	177.96 (17)
O2—C2—C3—C4	−178.93 (16)	C8—C9—C10—C4	178.22 (17)
C1—C2—C3—C4	−3.4 (3)	C1—C9—C10—C4	−2.3 (3)
O2—C2—C3—C11	4.0 (3)	O4—C4—C10—C5	−4.1 (3)
C1—C2—C3—C11	179.59 (18)	C3—C4—C10—C5	175.37 (17)
C2—C3—C4—O4	−173.37 (18)	O4—C4—C10—C9	176.20 (18)
C11—C3—C4—O4	3.8 (3)	C3—C4—C10—C9	−4.4 (3)
C2—C3—C4—C10	7.2 (3)	C2—C3—C11—C12	88.5 (2)
C11—C3—C4—C10	−175.65 (17)	C4—C3—C11—C12	−88.5 (2)
C10—C5—C6—C7	0.3 (3)	C3—C11—C12—C13	118.3 (2)
C5—C6—C7—C8	−1.1 (3)	C11—C12—C13—C15	178.2 (2)
C6—C7—C8—C9	0.6 (3)	C11—C12—C13—C14	−1.0 (4)
C7—C8—C9—C10	0.7 (3)	C2—O2—C16—O3	9.9 (3)
C7—C8—C9—C1	−178.78 (19)	C2—O2—C16—C17'	−170.81 (18)
O1—C1—C9—C8	4.2 (3)	C2—O2—C16—C17	−170.81 (18)
C2—C1—C9—C8	−174.46 (17)		

### Hydrogen-bond geometry (Å, º)

**Table:**

*D*—H···*A*	*D*—H	H···*A*	*D*···*A*	*D*—H···*A*
C11—H11*B*···O4^i^	0.97	2.55	3.274 (3)	131
C15—H15*A*···O1^ii^	0.96	2.59	3.485 (3)	156

Symmetry codes: (i) −*x*+1/2, *y*−1/2, −*z*+3/2; (ii) *x*+1/2, −*y*+1/2, *z*+1/2.
