# Supplementary material for: Crystal structure and Hirshfeld surface analysis of lapachol acetate 80 years after its first synthesis
Source: Acta Crystallogr E Crystallogr Commun. 2019 Aug 19;75(Pt 9):1362–6. doi: 10.1107/S2056989019011393 (PMC6727062; doi:10.1107/S2056989019011393)
Supplement: Supplementary file 3 [file e-75-01362-sup4.pdf]

Supporting Information

Crystal structure and Hirshfeld surface analysis of lapachol acetate 80 years after its first synthesis.

Miguel A. Martínez-Cabrera,[a] Mario A. Macías,[b] Francisco Ferreira,[c] Enrique Pandolfi,[d] Javier Barúa[e] and Leopoldo Suescun[f]*

[a]Universidad Nacional de Asunción, Facultad de Ciencias Exactas y Naturales, Departamento de Biología, Área Química Orgánica de los Productos, Naturales-LAREV, San Lorenzo Campus-UNA, Paraguay, [b]Department of Chemistry, Universidad de los Andes, Cra 1 N° 18A-12, 111711, Bogotá, Colombia, [c]Universidad Nacional de Asunción, Facultad de Ciencias Exactas y Naturales, Laboratorio de Análisis instrumental, Departamento de Química, San Lorenzo, Campus-UNA, Paraguay, [d]Laboratorio de Síntesis Orgánica, DQO, Facultad de Química, Universidad de, la, República, Montevideo 11800, Uruguay, [e]Universidad Nacional de Asunción, Facultad de Ciencias Químicas, San Lorenzo, Campus-UNA, Paraguay, and [f]Cryssmat-Lab/DETEMA, Facultad de Química, Universidad de la República, Av., Gral., Flores 2124, Montevideo 11800, Uruguay

Extraction of lapachol:

Lapachol was extracted from the wood of pink trumpet tree *Handroanthus heptaphyllus* (vell.) Mattos (locally called lapachol negro). 4 kg of ground wood was macerated exhaustively in dichloromethane for 30 days with daily stirring. After filtering the solid, lapachol was extracted selectively by deprotonation in a 5% sodium carbonate aqueous solution until complete discoloration of the organic phase. The aqueous solution containing deprotonated lapachol was slowly added with HCl 6N until complete precipitation of an intense yellow lapachol solid. The precipitate was filtered, washed with distilled water and dried. It was recrystallized from ethanol before using it for further synthesis. Yield 3.9%.

Synthesis of lapachol-acetate:

201 mg (0.823 mmol) of lapachol were dissolved in 5 mL of dry acetic anhydride and a catalytic amount of dry zinc chloride ($ZnCl_2$) were added. The suspension was refluxed for 30 minutes under an inert atmosphere (N_2). The solution was allowed to cool and 5 mL of glacial acetic acid and 50 mL of distilled water were added. The final mixture was refluxed for 10 minutes and allowed to stay overnight. The crude lapachol acetate was filtered, dried and purified by column chromatography (hexane: AcOEt, 9: 1 v/v) to obtain pure lapachol acetate as a yellow crystals (yield: 196 mg, 97.5%, m.p 79 °C) (lit. 79-80 °C) (Jacobsen, 1973). Adequate crystals were obtained for single-crystal X-ray diffraction analysis by dissolving the solid in a minimum amount of AcOEt in a rubber-stop vial in the center of which was inserted a syringe needle to facilitate slow evaporation of the solvent.

Lapachol acetate: **^{1}H NMR (400 MHz, $CDCl_3$) δ** 8.15-8.12 (m, 1 H), 8.12 - 8.09 (m, 1 H), 7.77 (dd, *J* = 6.4, 4.3 Hz, 1H), 7.74 (dd, *J* = 6.5, 4.5 Hz, 1H), 5.14 - 5.06 (m, 1H), 3.31 (s, 1H), 3.29 (s, 1H), 2.42 (s, 3H), 1.78 (s, 3H), 1.70 (s, 3H). (Figure S1) **IR (THF):** 2695, 2660, 2347, 2064, 1942, 1867, 1844, 1829, 1780, 1748, 1734, 1697, 1678, 1647,

1638, 1558, 1541, 1522, 1506, 1472, 1418, 1368, 1339,1294, 1271, 1190, 1173 cm^{-1}. **UV (EtOH, 0,164 mM):** 335, 270, 250, 205 nm. **UV (THF):** 332, 286, 282, 268, 250 nm. **GC-MS** (DB5, CH_2Cl_2): m/z 284 (M+), 269, 242, 227, 209, 199, 165, 105, 76. (Figure S2), **RT:** 29.8 minutes. **HPLC** (C18, FM: acetonitrile): **RT:** 4.035 minutes, **PP:** 100%. **Conductivity (THF, 1Mm, 22 °C):** 0.**00** µS.cm^{-1}. **Conductivity (DMSO-Biological assay medium, 1 Mm, 22 ° C):** 0.033 µS.cm^{-1}. **ε_{335} (EtOH, 0,164 mM)** = 4732 L. moL^{-1}. cm^{-1}, **ε_{270} (EtOH, 0,164 mM)** = 21582 L. moL^{-1}. cm^{-1}, **ε_{250} (EtOH, 0,164 mM)** = 28394 L. moL^{-1}. cm^{-1}, **ε_{205} (EtOH, 0,164 mM)** = 24988 L. moL^{-1}. cm^{-1}. **pH (EtOH, 25 °C, 0,164 mM):** 4.90.

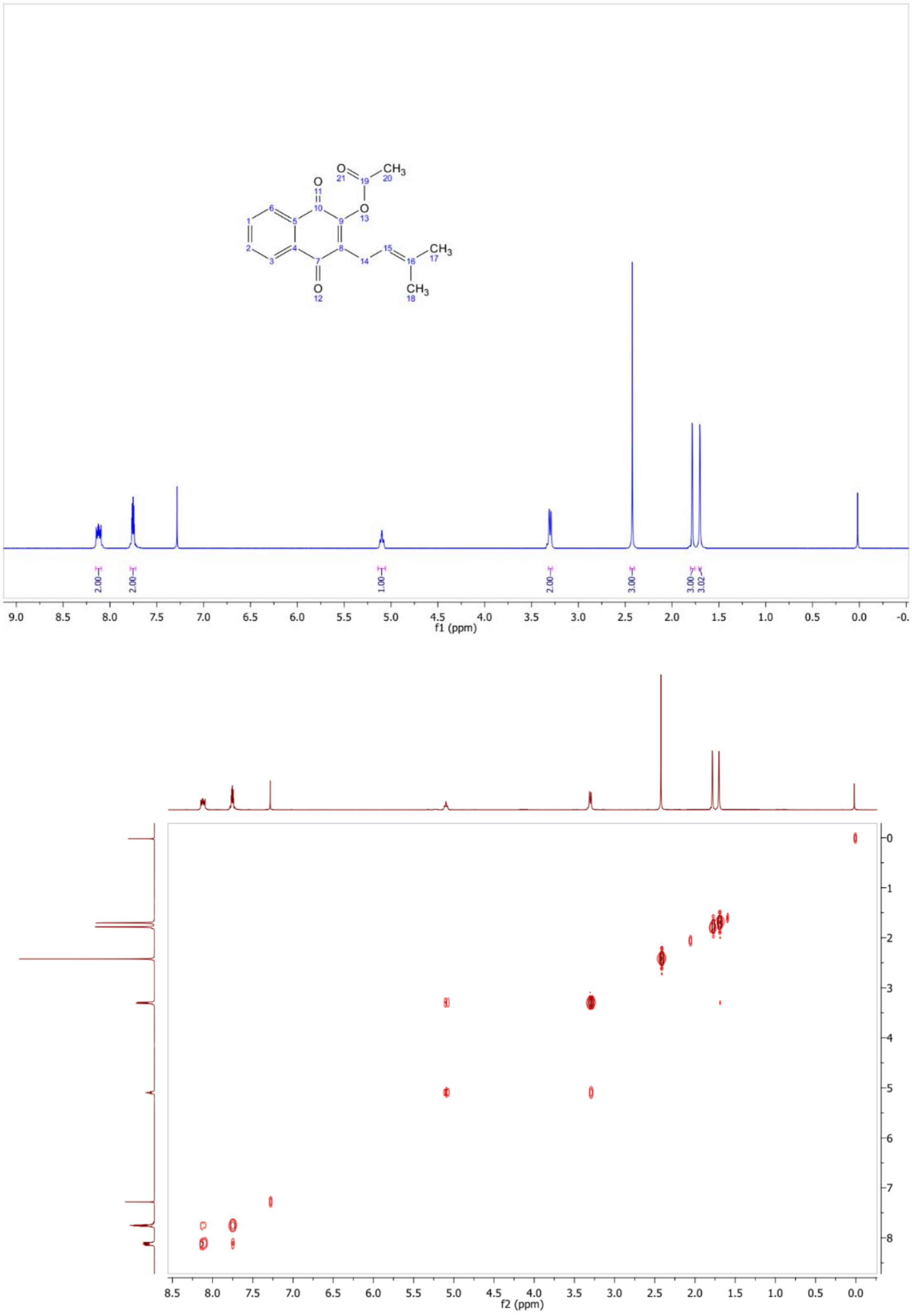

Figure S1: (a-top) ^{1}H-RMN and (b-bottom) COSY spectra of lapachol acetate.

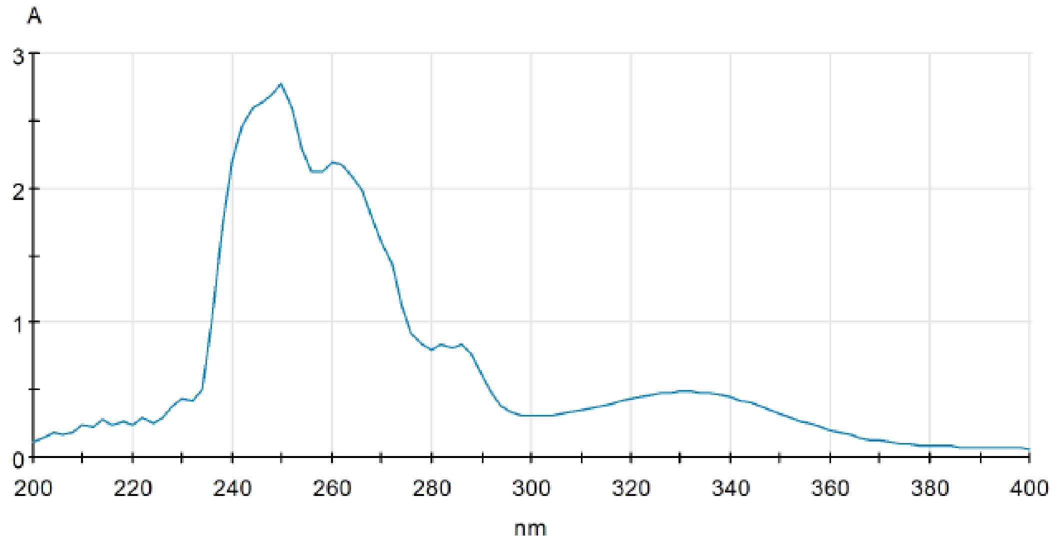

Figure S2. UV spectrum of lapachol acetate.

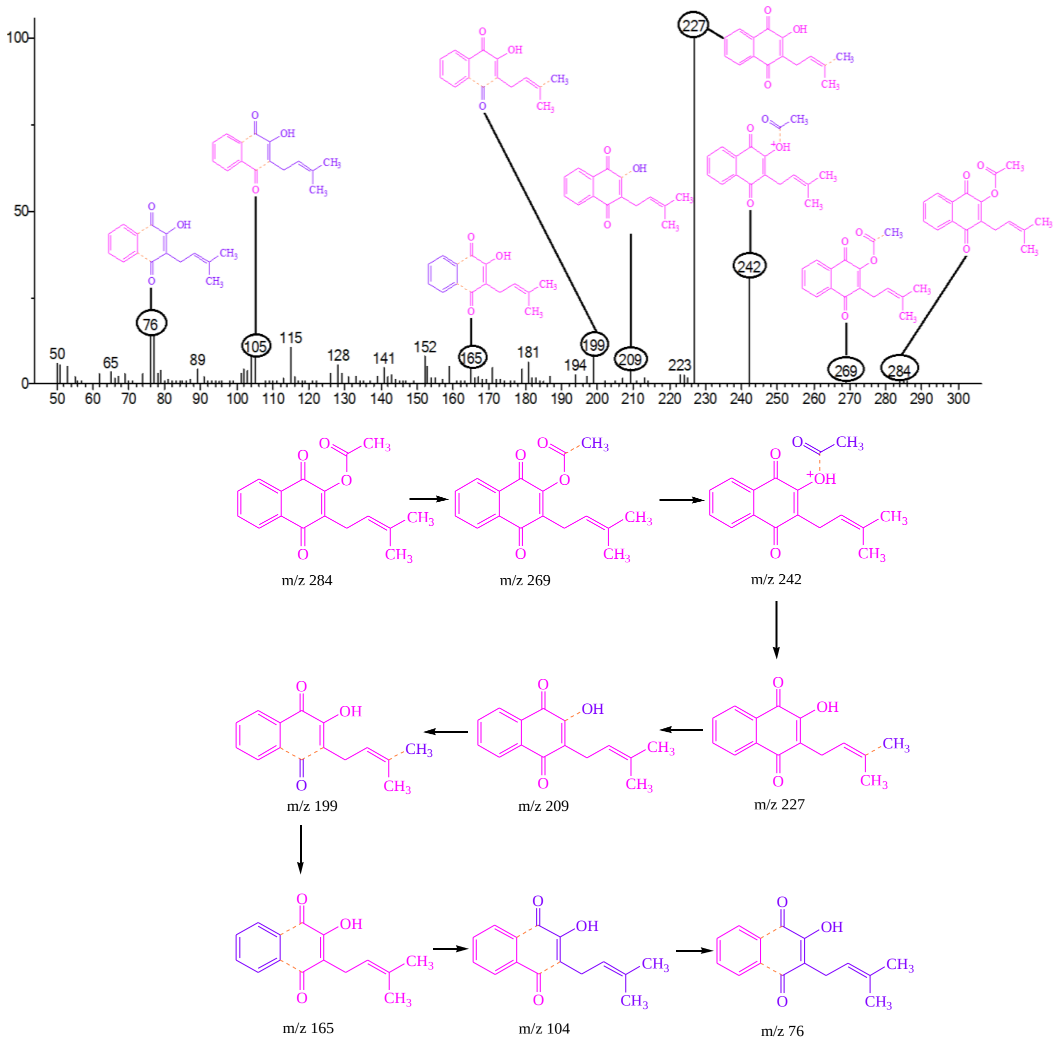

Figure S3. (a-top) Mass spectra of lapachol-acetate with the proposed decomposition fragments and (b-bottom) main breaking points and resulting fragments of lapachol-acetate after a mass spectrum.

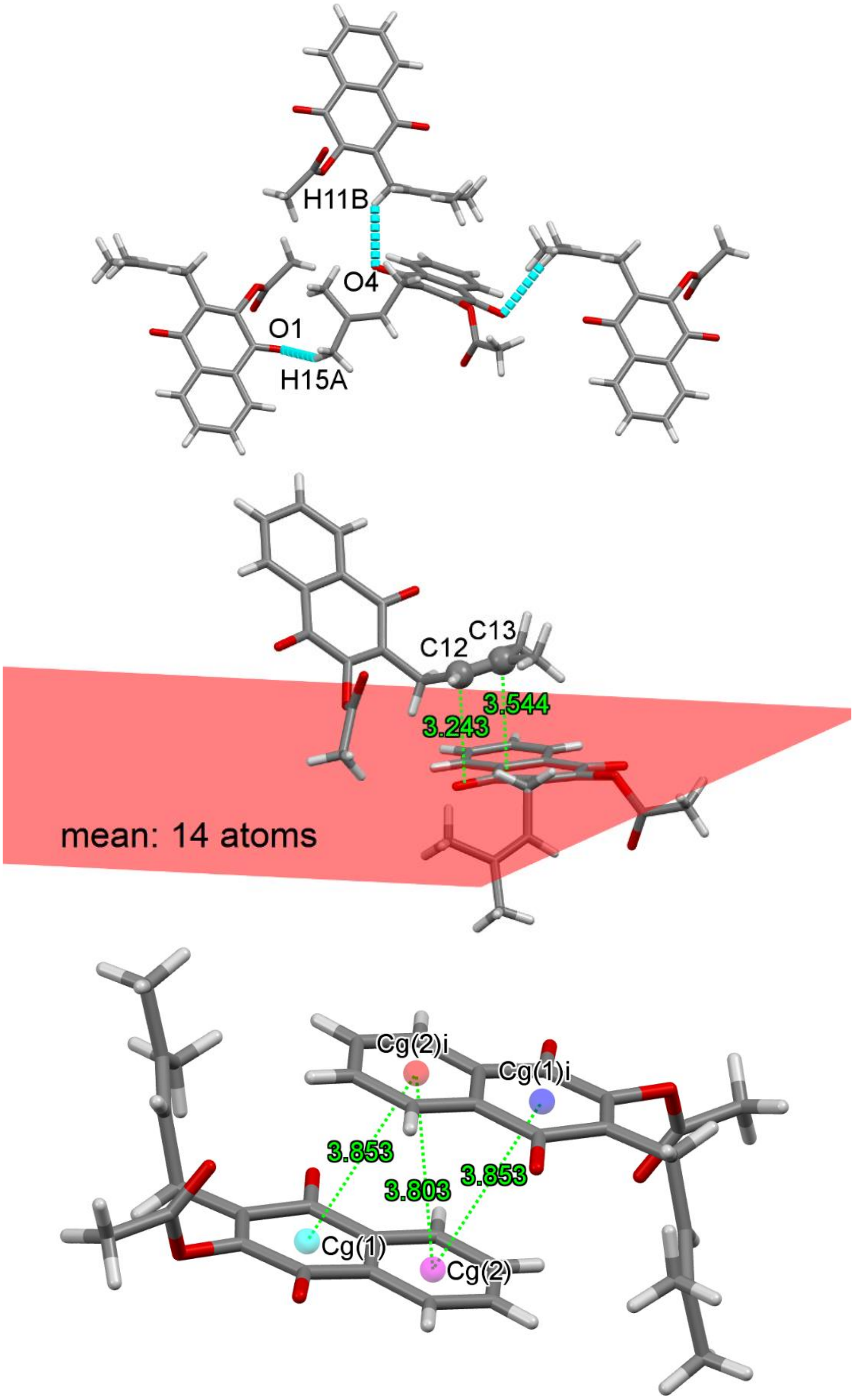

Figure S4: Main intermolecular interactions in lapachol acetate. (a-top) C-H…O interactions (b-center) butenyl-naphotoquinone π–π interaction and (c-bottom) Centroid-centroid distances between two inversion-related molecules

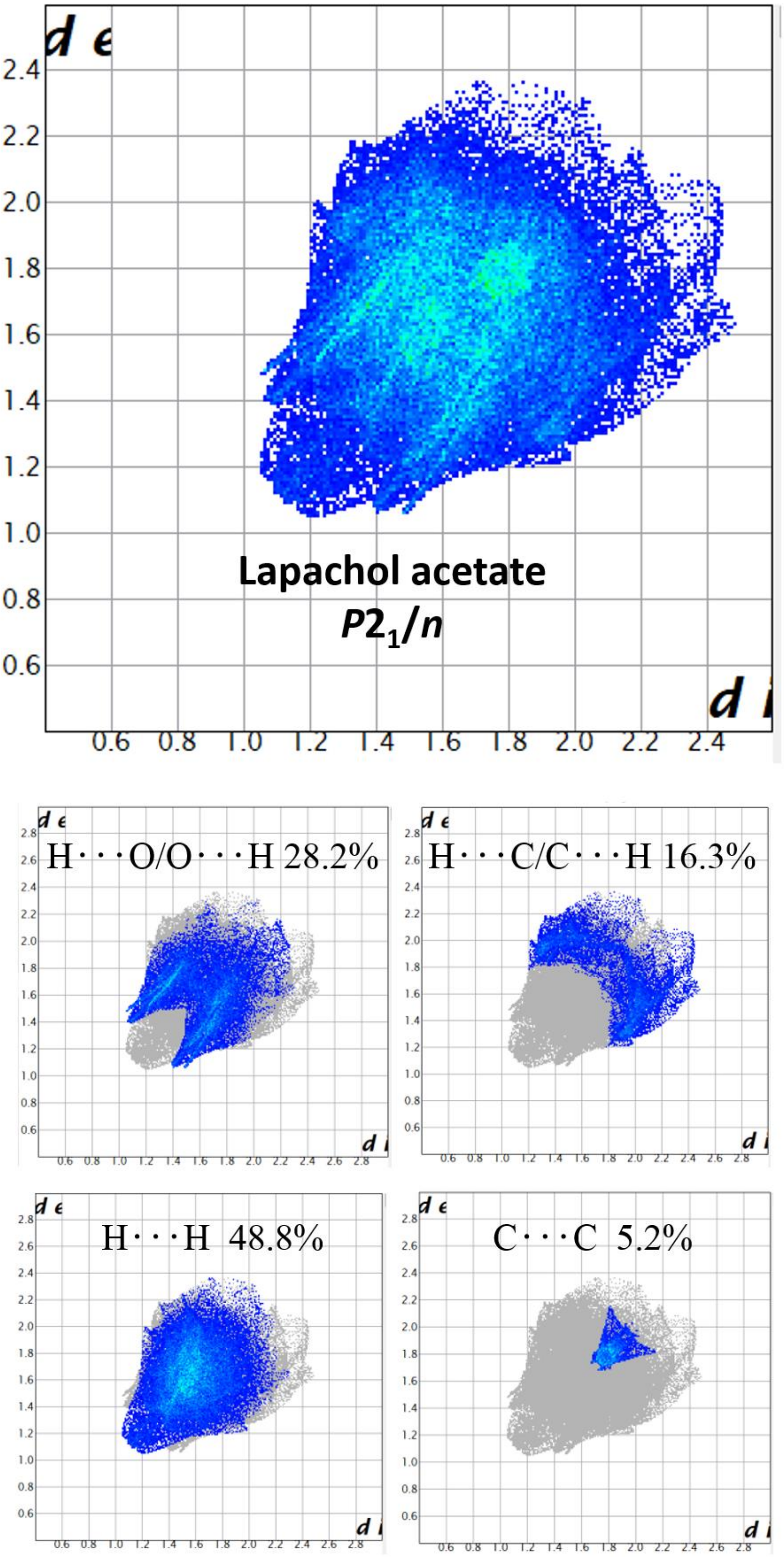

Figure S5: Two-dimensional fingerprints plots of lapachol acetate showing contributions from different types of contacts.

Table S1: CE-B3LYP interaction energies (kJ mol^{-1}) for lapachol acetate-RT[a]

Lapachol acetate-RT

C	*N*	*R*	E_{ele}	E_{pol}	E_{dis}	E_{rep}	E_{tot}
	1	9.74	-0.8	-0.3	-8.0	1.5	-7.1
	2	8.63	-8.3	-1.9	-21.3	16.7	-18.3
	2	7.03	-8.0	-1.2	-44.4	23.4	-33.6
	2	13.53	0.2	-0.1	-2.6	0.5	-1.9
	2	9.36	-5.9	-2.5	-14.3	6.9	-16.2
	1	7.39	1.3	-2.1	-10.1	1.8	-7.9
	2	9.47	-6.7	-1.7	-19.8	13.7	-17.2
	1	13.11	-1.1	-0.0	-9.0	4.8	-6.0
	1	5.97	-17.4	-3.0	-66.2	38.1	-54.7
	1	14.01	1.5	-0.2	-1.8	0.2	0.0

[a] N is the number of molecules with an R molecular centroid-to-centroid distance (Å) with an assigned C color-coded related to next Figure S6. Electron density was calculated using B3LYP/6-31G(d,p) model energies. Note: (*) scale factors used to determine E_{tot}: E_{ele} = 1.057; E_{pol} = 0.740; E_{dis} = 0.871; E_{rep} = 0.618.

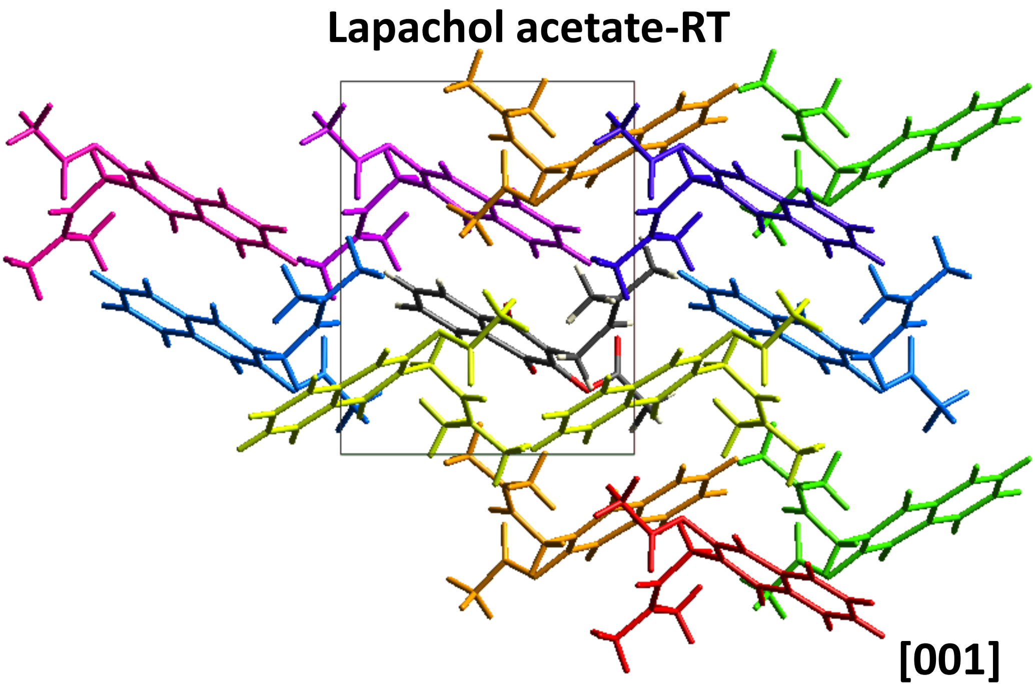

Figure S6: Colored representation of the centroid-to-centroid interactions observed in Table S2 of interaction energies. The molecule in black at the center corresponds to the asymmetric unit used as a reference for the interactions.
